# Mitochondrial Antioxidant SkQ1 Has a Beneficial Effect in Experimental Diabetes as Based on the Analysis of Expression of microRNAs and mRNAs for the Oxidative Metabolism Regulators

**DOI:** 10.3390/antiox10111749

**Published:** 2021-10-31

**Authors:** Yuliya Dvoretskaya, Victor Glanz, Mariya Gryaznova, Mikhail Syromyatnikov, Vasily Popov

**Affiliations:** 1 Laboratory of Metagenomics and Food Biotechnology, Voronezh State University of Engineering Technologies, 394036 Voronezh, Russia; dyd16@mail.ru (Y.D.); mariya-vg@mail.ru (M.G.); pvn@vsuet.ru (V.P.); 2 Department of Genetics, Cytology and Bioengineering, Voronezh State University, 394018 Voronezh, Russia; 3 BioME, LLC, 394026 Voronezh, Russia; viglanz@outlook.com

**Keywords:** mitochondria-targeted antioxidant, microRNA, oxidative stress, 10-(6′-plastoquinonyl)decyltriphenylphosphonium, qPCR, gene expression

## Abstract

Diabetes mellitus and related complications are among the most important problems of the world-leading healthcare systems. Despite their priority, molecular and genetic aspects of diabetes pathogenesis are poorly understood; however, the involvement of oxidative stress in this process is undoubted. Rats with experimental diabetes induced by the intraperitoneal injection of alloxan were subjected to the antioxidant pre-therapy with a series of mitochondria-targeted 10-(6’-plastoquinonyl)decyltriphenylphosphonium (SkQ1) injections and analyzed for the expression of mRNAs and microRNAs by real-time quantitative polymerase chain reaction to identify potential predictors of diabetes. Animals that received SkQ1 before diabetes induction demonstrated lower blood glucose levels compared to the diabetic animals not subjected to the therapy. SkQ1 caused changes in the mRNA levels of genes involved in the cellular defense against free radicals, which indicates a beneficial effect of the pre-therapy. Moreover, similar changes were observed on the epigenetic level, as the microRNA expression patterns not only proved the SkQ1 efficacy but also correlated with the expression levels of their mRNA targets. Oxidative stress and macromolecule damage by free radicals are determining factors in diabetes, which suggests that strategies aimed at restoring the antioxidant status of the cell can be beneficial. Mitochondria-targeted antioxidant SkQ1 demonstrates positive effects on several levels, from the normalization of the blood glucose content to genetic and epigenetic changes. Our results can serve as a basis for the development of novel therapeutic and diagnostic strategies.

## 1. Introduction

MicroRNAs are short non-coding RNAs (18–25 nucleotides in length) that are partially complementary to the regulatory regions in the 3′- or, more rarely, 5′-untranslated region (UTRs) of targeted mRNAs or even in their coding sequences [1]. The binding of microRNA inhibits translation of the target mRNAs or contributes to their degradation, eventually leading to gene silencing. MicroRNAs act as guides for positioning of the RNA-induced silencing complex (RISC), a molecular scaffold that facilitates interactions of microRNA with their targets, resulting in the inhibition of gene expression [2]. MicroRNAs form complex post-transcriptional regulatory networks that coordinate numerous cellular processes [3]. After the discovery of microRNAs in 1993, it has found that microRNAs affect the expression of at least 30% protein-coding genes, which makes microRNAs the most common regulators of gene expression [4]. However, only a small number of predicted microRNA-mRNA interactions have been experimentally confirmed. One microRNA can regulate many genes and vice versa; one gene can be regulated by several microRNAs [5].

Since even a small change in the microRNAs levels can affect the expression of hundreds of target genes and significantly change transcriptomes, the disruption of even a few microRNA regulatory networks is associated with the development of various diseases. It is believed expected that expression profiles that include microRNAs can be used for predicting the development of various diseases. The focus of many recent studies has been the identification of microRNAs involved in the pathogenesis of particular diseases, including diabetes. Aberrant microRNA expression can lead to significant impairments of glucose metabolism [6]. For example, overexpression of miR-375, which is the most abundant microRNA in pancreatic β-cells, reduces insulin secretion through the inhibition of myotrophin (MTPN) mRNA, downregulates expression of the 3′-phosphoinositide-dependent protein kinase 1 (PDK1) gene, and reduces transcription of the insulin gene [7,8,9]. At the same time, miR-375 is needed to maintain the required mass of β-cells [10]. It is known that microRNA-7 controls the maturation of pancreatic cells by interacting with the PAX6 mRNA, the increased levels of which promote differentiation of α- and β-cells [11].

Glucose homeostasis requires coordinated metabolic regulation of several tissues/organs, and microRNAs can play an important role in this communication [12]. Dysregulation of many microRNAs was observed in β-cells of diabetic animals, while restoration of normal levels of these microRNAs resulted in the improvement of cell sensitivity to insulin [13,14]. MicroRNAs are not only potential pharmacological targets for diabetes treatment but also promising clinical biomarkers for the early diagnosis of this disease [15].

Pancreatic β-cells are among the most metabolically active cells and are strongly dependent on oxidative metabolism for ATP synthesis, especially at elevated glucose concentrations [16]. The main source of ROS in pancreatic β-cells is the mitochondrial respiratory chain. Complexes 1 and 3, located in the inner mitochondrial membrane, generate superoxide ions via the single-electron reduction in molecular oxygen that cannot freely penetrate biological membranes [17]. Superoxide is converted to the less active H_2_O_2_ by superoxide dismutase isoenzymes. Hydrogen peroxide can diffuse through the membrane and transform into highly reactive hydroxyl radicals [18].

Potential harmful effects of elevated ROS production include oxidative damage to ribonucleic acids, proteins, and lipids. Therefore, ROS can affect the functioning and survival of β-cells via various mechanisms, such as changes in the enzymatic activity, dysregulation of gene expression, apoptosis, and others [19].

Since β-cells contain low amounts of antioxidant enzymes (superoxide dismutase, catalase, glutathione peroxidase), they are extremely sensitive to oxidative stress [20]. In this context, mitochondrial therapy and the use of mitochondria-targeted antioxidants, in particular, are gaining an increasing interest as potentially effective strategies for the prevention and treatment of many diseases, including diabetes.

SkQ1 is structurally similar to MitoQ, as they differ only in the antioxidant portion of the molecule (ubiquinone is replaced with plastoquinone). This modification is justified by the fact that plastoquinone is a more efficient antioxidant due to the unique properties of chloroplasts [21]. Therefore, ubiquinone replacement with plastoquinone should promote the antioxidant activity of the mitochondria-targeted compound and increase the “window” between the prooxidant and antioxidant effects [22].

Studying the effects of SkQ1 is a very promising approach since SkQ1 has proven to be more efficient than other types of mitochondria-targeted antioxidants in various studies [22].

It was demonstrated that SkQ1 can correct aberrations associated with age-related diseases, such as rheumatoid arthritis, osteoporosis, retinopathy, as well as accelerate wound healing (including diabetic lesions) [22,23]. The study published in 2014 reported that SkQ1 slows down oxidative hemolysis of erythrocytes [24]. Vnukov et al. demonstrated that SkQ1 administration in rats for five days significantly increased the mRNA levels of the transcription factor nrf2 and genes encoding SOD1, SOD2, CAT, and GPx4, which was accompanied by the activation of antioxidant enzymes (SOD, CAT, GPx, and GST) and increase in the concentration of reduced glutathione [25].

Our study is based on the assumption that SkQ1 has a positive impact on the development and course of experimental diabetes. The aim of this research was to study some genetic and epigenetic mechanisms of oxidative metabolism regulation in the pancreas of rats with experimental diabetes and to evaluate the efficacy of SkQ1 pharmacological intervention in the correction of damage caused by overproduction of free radicals.

## 2. Materials and Methods

### 2.1. The Object of Study

Male Wistar rats (*Rattus norvegicus*) weighing 250 ± 30 g were used in the study (6 animals in each group). The animals were kept on a standard rat chow with ad libitum access to food and water in a humidity- and temperature-controlled (21–22 °C) cages at a 12:12 h light/dark cycle (lights on: 07.00 h; lights off: 19.00 h).

Experimental type 1 diabetes mellitus (T1DM) was induced by a single intraperitoneal injection of 5% alloxan solution in 0.05 M sodium citrate (120 mg of alloxan per kg of animal body weight). The animals with blood glucose levels over 10 mmol/L were assigned to the experimental group. Blood glucose was measured with a OneTouch Select glucometer (LifeScan, Malvern, PA, USA). The dose of SkQ1 was selected by taking into account available experimental data and as a result of consultation with SkQ1 developers. It should be noted that for other model objects, the dose of the antioxidant should be adjusted [26,27,28]. A separate cohort of rats was injected with the same amount of SkQ1, and a diabetogenic dose of alloxan was administered to the animals on day 7 of the experiment.

### 2.2. Tissue Sampling

The rats were sacrificed by decapitation. Internal organs were perfused with 1× PBS buffer, after which the pancreas was immediately removed and placed on an ice-cold surface. RNALater™ reagent (Qiagen, Hilden, Germany) was injected with an insulin syringe throughout the entire tissue mass. Tissues processed in this way are characterized by the minimal RNA loss during extraction, as well as the high quality of the isolated RNA, suitable for subsequent manipulations.

### 2.3. Extraction of Total RNA

Total RNA was obtained by phenol-chloroform extraction with ExtractRNA ™ reagent (Evrogen, Moscow, Russia). Tissue samples were processed in a Dounce tissue grinder for 30 s, and RNA was isolated in accordance with the manufacturer’s protocol. Quantitative analysis and evaluation of RNA purity were carried out in a Hitachi U-2900 spectrophotometer (Hitachi, Tokyo, Japan); the absorbance of the RNA preparation was measured at 260 nm.

### 2.4. Reverse Transcription Reaction

RNA reverse transcription was performed in a Mastercycler Personal (Eppendorf, Hamburg, Germany) using an MMLV RT kit (Evrogen, Moscow, Russia).

### 2.5. Real-Time Polymerase Chain Reaction

Real-time quantitative polymerase chain reaction (qPCR) was performed with a CFX96 Real-Time PCR Detection System (Bio-Rad, California, USA) using qPCRmix-HS SYBR mix (Evrogen, Moscow, Russia) according to the manufacturer’s protocol. After amplification, the melting curves of the reaction products were analyzed in the temperature range from 65 to 95 °C. The primers were selected using Primer-BLAST (https://www.ncbi.nlm.nih.gov/tools/primer-blast; accessed on 28 September 2021). qPCR results were normalized to the expression levels of *gapdh* mRNA and 18S rRNA gene fragment. Primer sequences are presented in Table 1.

### 2.6. Isolation of RNA for Subsequent Analysis of microRNA

Total RNA was fractionated into long and short fractions using a Quick-RNA MiniPrep kit (Zymo Research, Irvine, CA, Cat No: R1054) according to the manufacturer’s protocol. The short RNA fraction was used as a template for reverse transcription.

### 2.7. MicroRNA Reverse Transcription

Reverse transcription was performed with miScript II RT Kit (Qiagen, Hilden, Germany, Cat No: 218160) as recommended by the manufacturer. Obtained cDNA was used as a template for the quantitative analysis of microRNA expression by qPCR.

### 2.8. qPCR

To detect mature microRNAs, we used miScript SYBR Green PCR Kit (Qiagen, Hilden, Germany, Cat No: 218073) with specific (forward) and universal (reverse) primers. Amplification was performed according to the following protocol: denaturation for 15 min at 95 °C; 40 cycles of denaturation for 15 s at 94 °C and annealing for 30 s at 55 °C; and final elongation for 30 s at 70 °C. The results of qPCR were normalized to the expression of microRNA RNU6B.

### 2.9. Ethical Expertise

The Ethical Committee for the Expertise of Biomedical Research at VSU “decided to unconditionally approve biomedical research on the topic «mitochondrial antioxidant SkQ1 has a beneficial effect in experimental diabetes based on quantitative expression analysis of the mRNA and microRNA oxidative metabolism regulator», responsible executor—Dvoretskaya Yuliya Dmitrievna, the results of the study can be recommended for publication” (excerpt from the protocol of conference No. 42–03 received on 26 November 2020, Ethics Committee for the Expertise of Biomedical Research at VSU).

### 2.10. Statistical Data Analysis

Results are shown as mean +/− standard error of the mean of three independent experiments conducted in three repeats. The data were analyzed with the Bio-Rad CFX Manager 3.1 software (Bio-Rad, CA, USA); relative gene expression was determined with the 2−ΔΔCt method. The groups were compared using the two-way Student’s *t*-test; *p*-value < 0.05 was considered statistically significant. Spearman’s ρ correlation coefficients were calculated.

## 3. Results

### 3.1. SkQ1 Pre-Therapy Normalizes Blood Glucose Level

**Figure 1:**
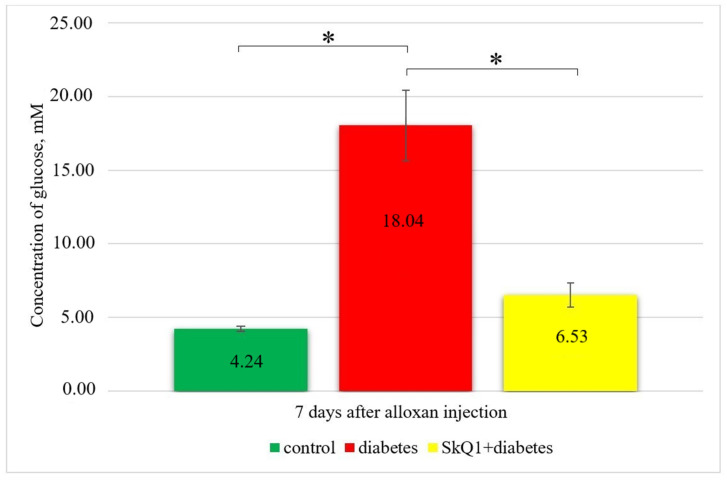
Effect of antioxidant therapy on hyperglycemia in experimental animals (* *p* ≤ 0.05).

Administration of diabetogenic doses of alloxan in rats resulted in the development of a stable model of insulin-dependent diabetes [29]. The effect of SkQ1 pre-therapy on the blood glucose concentration in rats with alloxan-induced diabetes is shown in Figure 1.

Rats that received preliminary SkQ1 injection displayed close to normal blood glucose levels (6.53 mM) compared to the control rats (4.24 mM), which can be explained by the fact that SkQ1 contributes to the normalization of blood glucose levels due to its protective properties.

### 3.2. SkQ1 Improves the Functioning of Cellular Defense System against Free Radicals by Affecting the Activity of Its Key Genes

**Figure 2:**
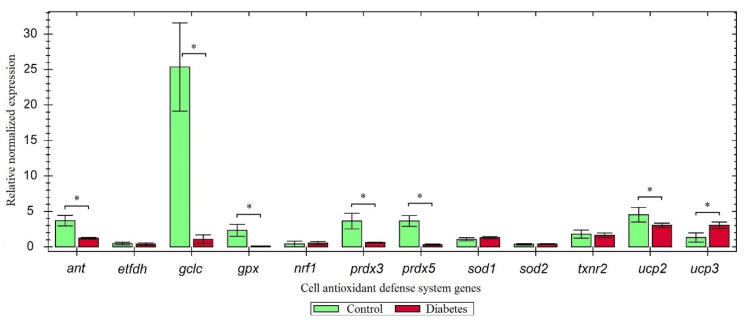
Expression pattern of genes-components of the cell antioxidant defense system in diabetes (* *p* ≤ 0.05).

We found that oxidative stress downregulated the expression of certain genes in animals with type 1 diabetes compared to the control rats (Figure 2).

The decrease in the gene expression could be related to a high content of oxidized proteins, lipids, and DNA in diabetic animals, which might reduce the ability of the cells to neutralize free radicals. In particular, the largest difference in the mRNA expression levels was observed for the *gclc* gene, which is presumably the reason for the decrease in the amount of glutathione in the cell. The downregulation of the *gpx*, *prdx3*, and *prdx5* genes encoding antioxidant proteins induces cellular susceptibility to oxidative stress, while the protein encoded by the *ant* gene plays an important role in the generation of ROS.

**Figure 3:**
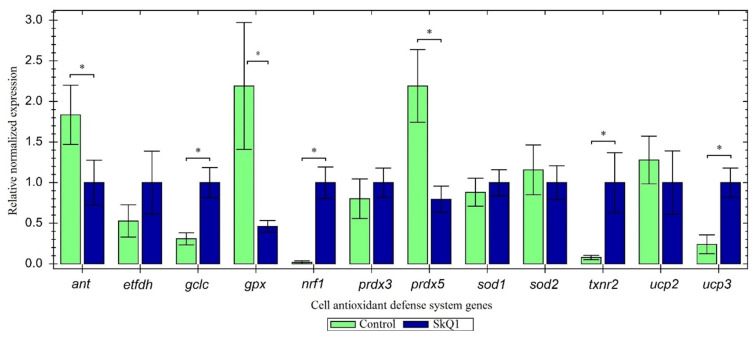
Effect of antioxidant SkQ1 on gene expression (* *p* ≤ 0.05).

SkQ1 exhibited no toxic effects in the organism. In some cases, it enhanced the cell’s enzymatic defense system against ROS; in other cases, it took on a significant portion of the protective functions (Figure 3).

SkQ1 injection increased the expression of the *gclc* gene and the gene for its transcription factor (*nrf1*). Apparently, SkQ1 takes on the main antioxidant function, which explains the downregulated expression of *prdx5* and *gpx*. It is interesting to note that SkQ1 injection also activated the system of peroxiredoxin regeneration (*txnr2*).

**Figure 4:**
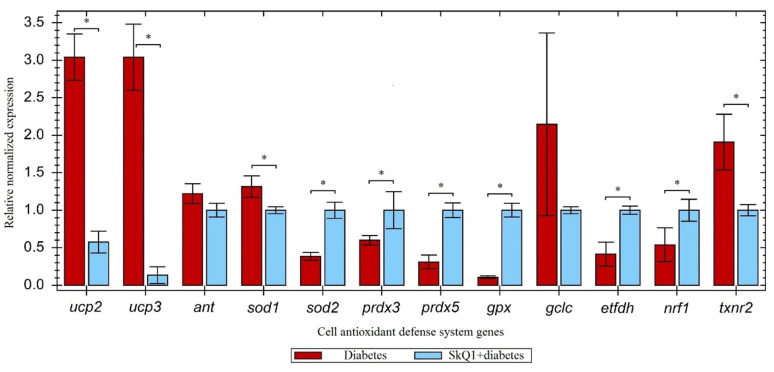
Efficiency of SkQ1 protective action against the background of developing type 1 diabetes mellitus (* *p* ≤ 0.05).

In order to understand the molecular mechanisms associated with the reduction in blood glucose levels following SkQ1 injection, we studied the expression of genes involved in oxidative metabolism in the pancreas (Figure 4).

Studying mRNA levels for the genes encoding enzymes of the cell antioxidant defense system, such as superoxide dismutase 2 (SOD2), electron transfer flavoprotein dehydrogenase (ETFDH), peroxiredoxin 5 (PRDX5), and glutathione peroxidase (GPX) showed that expression of these genes in the rats subjected to the SkQ1 pre-therapy was upregulated more than two times compared to the untreated diabetic rats. The increase in the expression of genes for peroxiredoxin 3 (PRDX3) and nuclear respiratory factor 1 (NRF1) was less than 2-fold, while expression of the SOD1 and TXNR2 genes was downregulated. The expression levels of mRNAs for the uncoupling proteins (UCP2, UCP3) were found to be greatly reduced after SkQ1 intervention. The expression level of the remaining genes displayed no statistically significant changes (see Discussion section for the discussion of the obtained results).

### 3.3. Expression Analysis of microRNA Target Genes

**Figure 5:**
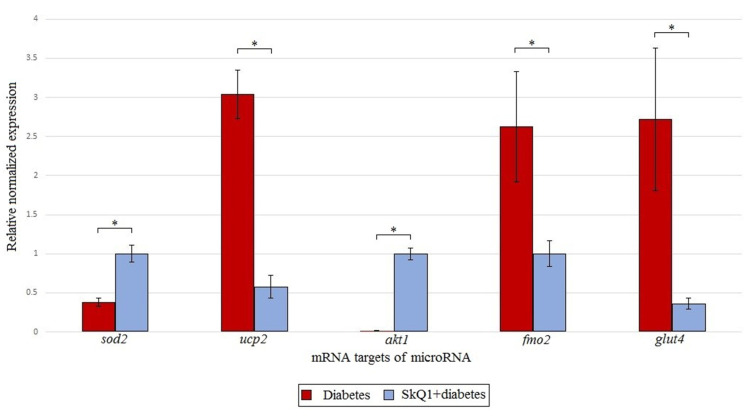
Expression analysis of mRNA targets of microRNAs after pre-therapy with SkQ1 (* *p* ≤ 0.05).

Targeted mRNAs were identified based on the analysis of the miRDB (http://mirdb.org; accessed on 28 September 2021) and miRBase (https://mirbase.org; accessed on 28 September 2021)) databases. Figure 5 shows changes in the expression of the microRNA target genes involved in the T1DM pathogenesis.

Figure 5 also shows results for other genes that are involved in the pathogenesis of diabetes and perform other important functions. It is interesting to note that despite the fact that GLUT4 transports glucose into the cell, SkQ1 greatly reduced the content of mRNA for this protein.

### 3.4. MicroRNAs as Biomarkers of Type 1 Diabetes Mellitus

**Figure 6:**
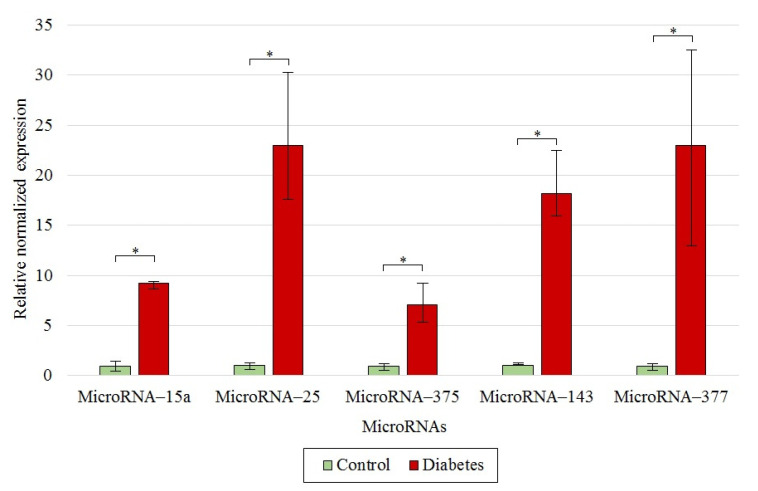
Expression analysis of microRNAs in type 1 diabetes (* *p* ≤ 0.05).

We have also evaluated the expression of microRNAs in the pancreatic tissue of rats from the experimental and control groups. Upregulated expression of microRNA-143, -25, -377, -375, and -15a in the diabetic rats (Figure 6) could indicate the association between expression of these molecules and disease development.

### 3.5. Analysis of Expression of microRNAs and Their Target Genes

**Figure 7:**
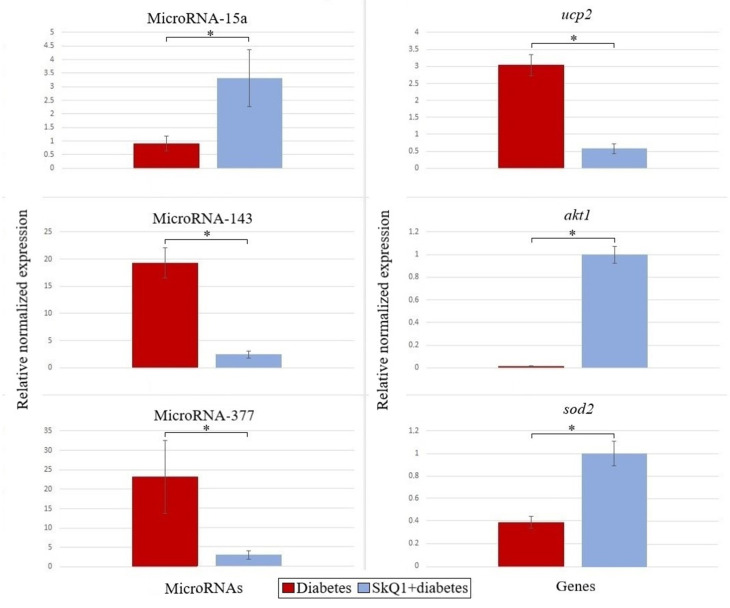
Effect of SkQ1 pre-therapy on the levels of various microRNAs and expression of their target genes (* *p* ≤ 0.05).

We studied the antioxidant status of the cells at two levels (genetic and epigenetic) and found that in a number of cases, expression of microRNAs correlates with the expression of their mRNA targets, which suggests the existence of the possible mechanism for the regulation of gene expression (Figure 7).

Injection of SkQ1 changed the expression of some microRNAs in the process of diabetes development compared with the levels of the same microRNAs in the pancreas of rats that received alloxan only. All microRNAs studied in our work displayed almost normal levels in the diabetic rats pretreated with SkQ1. It should be noted that the level of miR-15a in the diabetic rats was 3.6 times lower than in the animals subjected to the SkQ1 pre-therapy.

## 4. Discussion

The commonly known function of UCP2 is proton gradient dissipation and subsequent conversion of ATP energy into heat; however, another is inhibition of glucose-stimulated insulin secretion [30]. An increase in the ATP/ADP ratio results in the closing of the ATP-sensitive potassium channel in the inner mitochondrial membrane. This causes membrane depolarization, opening of calcium channels, and influx of Ca^2+^ into the cytosol of β-cell, leading to the exocytosis of insulin-containing granules [31]. Due to its ability to increase proton leakage, UCP2 reduces ATP production through the glucose metabolism in β-cells and, therefore, impairs glucose-stimulated insulin secretion [32]. Conversion of ATP energy into heat is a protective mechanism, which explains upregulated *ucp2* expression in the control group compared with the diabetic rats (Figure 2). However, at the same time, UCP2 inhibits insulin secretion, which is manifested as an elevated content of *ucp2* mRNA in rats with alloxan-induced diabetes vs. the diabetic group treated with SkQ1 (Figure 4). Supposedly, the UCP2-dependent proton flux activated by oxidative stress can be pharmacologically inhibited. This should increase insulin secretion and prevent β-cell dysfunction, which explains the inhibitory effect of SkQ1 on the expression of *ucp2*.

Uncoupling protein 3 (UCP3) is involved in fatty acid metabolism and energy homeostasis; it also modulates cell sensitivity to insulin [33]. It is believed that UCP3 participates in the energy expenditure via uncoupling, especially in the metabolism of fatty acids, and presumably protects mitochondria from the oxidative stress induced by lipid peroxidation [34]. Several authors suggested that UCP3 contributes to the reduction in ROS generation, either indirectly or directly [35,36]. After SkQ1 pre-therapy, this protective function of UCP3 is delegated to SkQ1, probably, because SQ1 is a more efficient antioxidant. As a result, *ucp3* mRNA is synthesized in smaller amounts (Figure 4).

The thioredoxin system is involved in the neutralization of ROS by transferring electrons to various peroxidases. The oxidized form of thioredoxin is reduced by thioredoxin reductase [37]. We found that SkQ1 failed to normalize the level of mitochondria-specific thioredoxin reductase 2 (*txnr2*) mRNA (Figure 4). Apparently, SkQ1 plays the main role in antioxidant protection, which explains the decrease in the expression of the peroxidase regeneration system proteins.

As a result of increased ROS generation, SOD converts superoxide into hydrogen peroxide, thus facilitating H_2_O_2_ decomposition to water and oxygen by other enzymes (catalase, glutathione peroxidase, and peroxiredoxins) [38]. Manganese-containing SOD2, which is located in the mitochondrial matrix, catalyzes dismutation of superoxide anion radicals to hydrogen peroxide, thereby protecting iron/sulfur-containing mitochondrial enzymes from exposure to superoxide [39]. Pre-therapy with SkQ1 significantly upregulated the expression of *sod2* (Figure 4.). Superoxide dismutase 1 (SOD1) is responsible for the use of superoxide in the cytosol [38]. However, there is evidence that SOD1 may also act as a nuclear transcription factor to control the overall response to oxidative stress [40]. We hypothesize that the decrease in the SOD1 expression in the diabetic mice injected with SkQ1 could be due to the ability of SkQ1 to reduce the level of oxidative stress, leading to the reduction in the SOD1 role as a transcription factor.

Peroxiredoxin 3 (PRDX3) plays an important role in preventing apoptosis induced by oxidative stress in β-cells. PRDX3 is the main mitochondrial peroxiredoxin that removes ROS using thioredoxin 2 as a physiological electron reducing agent [41]. Peroxiredoxin 5 (PRDX5) decreases the levels of hydrogen peroxide, peroxynitrite, and alkyl hydroperoxide [42]. Reduced expression of *prdx3* and *prdx5* promotes cell susceptibility to oxidative damage (Figure 2). Overexpression of peroxiredoxin genes caused by SkQ1 pre-therapy inhibits peroxide accumulation and decreases the risk of apoptosis (Figure 4).

Nuclear respiratory factor 1 (NRF1) acts as a transcription factor and activates the expression of several genes involved in the regulation of cell growth and mitochondrial respiration, as well as in mitochondrial biogenesis [43]. Injection of mitochondrial-targeted antioxidants can increase the expression level of this transcription factor (Figure 4).

GPX (glutathione peroxidase) is a well-known selenium enzyme that protects cells from oxidative stress via catalytic reduction in harmful peroxides. Multiple studies have demonstrated that the expression of *gpx* mRNA is reduced in T1DM [44,45]; we also observed this effect in our study (Figure 2). Figure 4 demonstrates upregulation of the GPX mRNA level, which indicates activation of the cell antioxidant defense in response to the excess of free radicals.

The protein product of the *gclc* gene (the catalytic subunit of glutamate-cysteine ligase) is a regulator of glutathione level in the cell. Insulin deficiency is known to reduce the expression of *gclc* (as confirmed in our research, Figure 2) and, consequently, the expression of *gsh*, whose protein product is a multifunctional antioxidant [46]. The SkQ1 pre-therapy failed to upregulate the expression of this enzyme (Figure 4).

Electron transfer flavoprotein dehydrogenase (ETFDH) plays an important role in the β-oxidation of fatty acids. This enzyme oxidizes electron-transporting flavoprotein (ETF) and transfers electrons to ubiquinone, thus reducing it to ubiquinol, which has antioxidant properties [47]. Therefore, it can be assumed that under conditions of acute oxidative stress, SkQ1 promotes the synthesis of another natural antioxidant by functioning as a transcription factor (Figure 4).

Adenine nucleotide transporter (ANT) provides a continuous supply of ADP required to support the process of oxidative phosphorylation. The exchange of ADP to ATP by ANT plays a significant role in maintaining the activity of ATP synthase and normal levels of the mitochondrial transmembrane potential (Δψ). Disruption of the ANT activity decreases mitochondrial synthesis of ATP and increases Δψ. In the cells with weak ATP/ADP metabolism, ANT plays an important role in the generation of ROS and induction of apoptosis [48].

Serine/threonine protein kinase (*akt1* gene product), also called protein kinase B, is a direct downstream target of phosphoinositide 3-kinase (PI3K) of the PI3K/AKT/mTOR pathway, which is activated by many growth factors and insulin and plays a key role in the regulation of cell cycle, cell survival, and glucose metabolism [49]. It was demonstrated that transgenic mice expressing constitutively active AKT (CA-AKT1) in β-cells show a significant increase in the mass of Langerhans islets, better glucose tolerance, and resistance to experimentally induced diabetes [50]. Activation of AKT1 by various stimuli has been shown to promote the survival and proliferation of islet cells. All these data indicate that AKT1 may play a therapeutic role in diabetes development, as confirmed by the upregulation of the *akt1* gene expression by the SkQ1 pre-therapy (Figure 5).

The protein product of the *fmo5* gene (flavin-containing monooxygenase) can function as a NADFH oxidase, producing hydrogen peroxide [51]. Therefore, it is reasonable to expect an increase in the expression of this protein under conditions of acute oxidative stress. SkQ1 suppressed expression of *fmo5* level (Figure 5), thereby decreasing ROS production.

*Glut4* encodes a type 4 glucose transporter that mediates insulin-dependent glucose transport across the cell membrane [52]. Dysfunction of this protein leads to insulin resistance. However, recently, Khelifi et al. revealed the relationship between GLUT4 and death-associated protein 6 (DAXX) that promotes cell death under oxidative stress [53]. Therefore, it can be assumed that SkQ1 prevents the development of conditions leading to apoptosis (Figure 5).

The next stage of the study was to estimate the effect of SkQ1 pre-therapy on the expression of various microRNAs.

Several microRNAs are known to regulate the natural processes of insulin biosynthesis and its secretion by pancreatic β-cells. For example, microRNA-375 and -15a were among the first microRNAs described as key factors in the regulation of insulin secretion [9,54]. It is believed that the level of microRNA-25 correlates with the residual function of β-cells and with an adequate glycemic control [55]. Overexpression of microRNA-143 impairs insulin sensitivity by affecting proteins involved in the maintenance of lipid composition of the membranes [15]. SkQ1 pre-therapy led to the normalization of glucose metabolism, which was manifested as downregulation of microRNA-143 expression (Figure 7). Evaluation of the expression of such microRNAs might be used to distinguish between T1DM patients and healthy individuals, i.e., these microRNAs can serve as early biomarkers of T1DM.

Expression of microRNA-15a, which positively regulates insulin biosynthesis by inhibiting *ucp2* expression, was increased after SkQ1 pre-therapy against the background of suppressed *ucp2* expression, which indicated the efficacy of the therapy. Similarly, we observed an inverse relationship between the levels of microRNA-377 and *sod2* mRNA, as well as between microRNA-143 and *akt1* mRNA (Figure 7). No relationship was found between the other studied microRNAs and their putative target genes.

## 5. Conclusions

In this study, we evaluated expression patterns of genes involved in the enzymatic defense against ROS in the pancreatic β-cells of rats with the experimentally induced T1DM. We found that SkQ1 promoted normalization of blood glucose levels in the animals with the developed insulin-dependent diabetes. The ability of SkQ1 to act as a protective antioxidant and to restore the natural mitochondrial defense systems under conditions of acute oxidative stress has been demonstrated. We also studied the expression levels of various microRNAs that affect the functioning and survival of β-cells and are involved in insulin biosynthesis. The observed changes in the expression of selected microRNAs suggest that analysis of expression of these molecules can be used for the prognosis and diagnostics of T1DM. Our results showed that preliminary administration of SkQ1 had a positive effect on the expression of microRNA markers determining the functional activity of β-cells. In addition, an inverse relationship was shown for the expression levels of microRNA-15a, -377, and -143 and their target genes (*ucp2*, *sod2*, and *akt1*, respectively).

The obtained data suggest that SkQ1 is an efficient regulator of cellular response to oxidative stress and a potential tool for the correction of pathological conditions associated with excessive ROS production. The data on the protective role of SkQ1 in experimentally induced diabetes may contribute to the further development of pharmacological preparations. In addition to the identification of biomarkers, analysis of the microRNA expression patterns might help to better understand the biology of β-cells and the molecular processes underlying the development of diabetes.

## Figures and Tables

**Table 1:** Primer sequences.

Abbreviation	Forward	Reverse
*gapdh*	5′-GGGGCTCTCTGCTCCTCCCTGT-3′	5′-CATGGGGGCATCAGCGGAAGG-3′
*18S rRNA*	5′-CGGCTACCACATCCAAGCAA-3′	5′-GCTGGAATTACTGTGGCT-3′
*ucp2*	5′-ATGGTTGGTTTCAAGGCCACA-3′	5′-CGGTATCCAGAGGGAAAGTGAT-3′
*ucp3*	5′-CTGCACCGCCAGATGAGTTT-3′	5′-ATCATGGCTTGAAATCGGACC-3′
*prdx3*	5′-GGTTGCTCGTCATGCAAGTG-3′	5′-CCACAGTATGTCTGTCAAACAGG-3′
*prdx5*	5′-CAGCTGAGGTTTTGCGTCCT-3′	5′-CCAGGCAGATGGGTCTTGGA-3′
*etfdh*	5′-GTGCGACTAACCAAGCTGTC-3′	5′-GGATGAACAGTGTAGTGAGTGG-3′
*sod1*	5′-AACCAGTTGTGTTGTCAGGAC-3′	5′-CCACCATGTTTCTTAGAGTGAGG-3′
*sod2*	5′-CAGACCTGCCTTACGACTATGG-3′	5′-CTCGGTGGCGTTGAGATTGTT-3′
*txnr2*	5′-GATCCGGTGGCCTAGGTTG-3′	5′-TCGGGGAGAAGGTTCCACAT-3′
*ant*	5′-AGCTCCCGGATCCCAAGAAT-3′	5′-GCATCATCATACGACGGCGA-3′
*gclc*	5′-GGGGTGACGAGGTGGAGTA-3′	5′-GTTGGGGTTTGTCTTCTCCC-3′
*gpx*	5′-AGTCCACCGTGTATGCCTTCT-3′	5′-GAGACGCGACATTCTCAATGA-3′
*nrf1*	5′-AGCACGGAGTGACCCAAAC-3′	5′-TGTACGTGGCTACATGGACCT-3′
*fmo5*	5′-CTCTGCCAGGAGTTGTAGCC-3′	5′-CTTTTCTTGGCCATGGTCGC-3′
*akt1*	5′-GTGGCAAGATGTGTATGAG-3′	5′-CTGGCTGAGTAGGAGAAC-3′
*glut4*	5′-GCCGGGACACTATACCCTA-3′	5′-CCCAGCCAAGTTGCATTGTAG-3′

## Data Availability

Data is contained within the article.
